# Nutrigerontology: why we need a new scientific discipline to develop diets and guidelines to reduce the risk of aging-related diseases

**DOI:** 10.1111/acel.12284

**Published:** 2014-12-03

**Authors:** Kris Verburgh

**Affiliations:** Center Leo Apostel for Interdisciplinary Studies (CLEA), Free University of Brussels (VUB)Brussels, Belgium

**Keywords:** aging, aging-related diseases, biogerontology, cardiovascular disease, diabetes type 2, diet, nutrient-sensing pathways, nutrition, obesity, overweight

## Abstract

Many diets and nutritional advice are circulating, often based on short- or medium-term clinical trials and primary outcomes, like changes in LDL cholesterol or weight. It remains difficult to assess which dietary interventions can be effective in the long term to reduce the risk of aging-related disease and increase the (healthy) lifespan. At the same time, the scientific discipline that studies the aging process has identified some important nutrient-sensing pathways that modulate the aging process, such as the mTOR and the insulin/insulin-like growth factor signaling pathway. A thorough understanding of the aging process can help assessing the efficacy of dietary interventions aimed at reducing the risk of aging-related diseases. To come to these insights, a synthesis of biogerontological, nutritional, and medical knowledge is needed, which can be framed in a new discipline called ‘nutrigerontology’.

## Introduction

Great progress has been made in the course of recent decades in the field of aging research. Biogerontology, the science that studies aging, uncovered some important molecular pathways that are major modulators of the aging process (Kenyon, 2010; Fontana *et al*., 2010; Bartke *et al*., 2013). At the same time, society is confronted with an ever-increasing rise in the prevalence of aging-related diseases, such as cardiovascular disease, type 2 diabetes, and Alzheimer's disease. Extending knowledge from biogerontology to clinical medicine and nutritional science can help discover adequate dietary recommendations to prevent or slow down the progress of various aging-related diseases. This knowledge can be framed into ‘nutrigerontology’, a new scientific discipline that encompasses biogerontology, medicine, and dietetics. Nutrigerontology researches how food substances, foods, food patterns, and diets influence the risk of aging-related diseases and (healthy) lifespan. Nutrigerontology can also be considered as a practical application of biogerontology. This article wants to clarify how biogerontological insights can be used to create dietary recommendations that can reduce the risk of various aging-related diseases that are on the rise in both developed and developing countries.

## Reduced insulin/IGF-1 and mTOR signaling increases lifespan

In the field of biogerontology, the two most well-known pathways implicated in the aging process are the insulin/insulin-like growth factor signaling pathway (IIS pathway) (Bartke *et al*., 2013) and the mammalian or mechanistic target of rapamycin (mTOR) pathway (Johnson *et al*., 2013). We will initially focus on these two canonical pathways to show how a better understanding of the aging process can help to develop better long-term diet recommendations. The mTOR and IIS pathways are nutrient-sensing pathways, implying that they are activated by nutrients that we eat, such as carbohydrates (which mainly activate the IIS pathway, but also the mTOR pathway) (Bartke *et al*., 2013) and amino acids (which mainly activate the mTOR pathway, but also the IIS pathway) (Wullschleger *et al*., 2006). The IIS pathway exerts its effects through transmembrane insulin and IGF-1 receptors, which initiate glucose uptake in the cell, and stimulate cell growth and cell proliferation. The mTOR protein is intracellularly located and a potent stimulator of protein translation.

A mounting body of research shows that loss of function of the IIS pathway and mTOR pathway increases lifespan. Modulating these pathways, genetically or nutritionally, can impact the rate of aging and postpones the advent of aging-related diseases.

For example, mice with a fat-specific insulin receptor knockout (FIRKO) genotype live 18% longer (Blüher *et al*., 2003) and reducing insulin receptor signaling in the mouse brain extended lifespan up to 18% (Taguchi *et al*., 2007). Homozygous deletion of the insulin receptor substrate 1 (Irs1-/-), an effector of the insulin receptor, increased median lifespan by 32% in female mice. However, deletion of IRS-1 in male mice did not lead to an increased lifespan (Selman *et al*., 2008). Mice heterozygous for the IGF-1 receptor (Igf1r+/-) live on average 26% longer than their wild-type littermates (Holzenberger *et al*., 2003). In humans, the insulin/insulin-like growth factor signaling pathway also is involved in lifespan regulation. Polymorphic variants of IGF-related pathways confer health and lifespan benefits in humans (Bonafè *et al*., 2003; Kojima *et al*., 2004; Pawlikowska *et al*., 2009). Female Ashkenazi Jewish centenarians show an overrepresentation of loss-of-function mutations in the IGF-1 receptor (Suh *et al*., 2008). Laron dwarves, who are growth hormone receptor deficient, have reduced levels of IGF-1, and despite being generally overweight, they have a major reduction in the risk of type 2 diabetes and cancer (Guevara-Aguirre *et al*., 2011; Steuerman *et al*., 2011). People with acromegaly (due to increased growth hormone and IGF-1 production) have a two- to threefold increase in mortality, mostly because of vascular disease (Clayton, 2003).

Regarding the mTOR pathway, loss-of-function of mTOR or proteins involved in the mTOR pathway doubles the lifespan in the nematode *Caenorhabditis elegans* (Vellai *et al*., 2003) and extends lifespan in the fruit fly *Drosophila melanogaster* (Kapahi *et al*., 2004). Mice that express mTOR at 25% of wild-type mice display a 20% increase in median lifespan (Wu *et al*., 2013). Rapamycine, a pharmacological mTOR inhibitor, extends lifespan with 9% in male and 14% in female mice (Harrison *et al*., 2009).

## The IIS and mTOR pathways in aging-related disease

How do the IIS and mTOR pathway contribute to the aging phenotype? A continuous stimulation of the IIS and mTOR pathways leads to a higher risk of aging-related diseases and reduced lifespan via reduced autophagy (less clearance of protein aggregates and cellular organelles), increased protein agglomeration and proteotoxicity (mTOR activation leads to increased protein production and reduced protein clearance), inflammation, reduced expression of antioxidant proteins, mitochondrial dysfunction, and other mechanisms (Kenyon, 2010; Fontana *et al*., 2010; Johnson *et al*., 2013; Cuervo, 2008). These mechanisms increase the risk of insulin resistance (Shah *et al*., 2004)*,* atherosclerosis (Martinet *et al*., 2014), cardiomyopathy (Willis & Patterson, 2013), neurodegenerative diseases (O'Neill *et al*., 2012, 2013), cancer (Martini *et al*., 2014), osteoporosis (Glantschnig *et al*., 2003) and other aging-related diseases and symptoms.

One of the most intriguing aspects of the IIS and mTOR pathways is that these are *nutrient*-sensing pathways, implying that they are activated by nutrients (carbohydrates and amino acids), which are contained in the food we eat. An overabundance of these nutrients can accelerate the aging process and increase the risk of aging-related diseases such as cardiovascular disease and type 2 diabetes. This fits in a new emerging paradigm shift, in which the aging process is viewed as a consequence of continuous cellular stimulation and hyperfunction in adulthood, in which a continuous bombardment of our cells with nutrients, growth factors, and mitogenic stimuli accelerates the aging phenotype (Gems & de la Guardia, 2012; Blagosklonny, 2006).

Extrapolating knowledge from the biogerontological field to nutritional science and medicine could lead to a new scientific field, called ‘nutrigerontology’. A diet that reduces the risk of aging-related diseases could be one that delivers carbohydrates, amino acids, and fats in such a way that pro-aging nutrient-sensing pathways such as the IIS and mTOR pathway are less activated. The ideal composition of such a diet remains to be determined, but animal and human studies already provide interesting clues.

## mTOR, diets, and nutrition

Because aging-related pathways like mTOR are activated by amino acids, a diet that does not provide an overabundance of amino acids can be considered beneficial (Efeyan *et al*., 2012). Restricting proteins is one of the few dietary interventions that can increase lifespan in rodents (Yu *et al*., 1985; Leto *et al*., 1976; Ross, 1961; Ross & Bras, 1975). Mice on low-protein diets (5% to 15% protein) live up to 150 weeks compared to 100 weeks for mice on a high-protein diet (50% protein), and this increase in lifespan was associated with reduced mTOR activation (Solon-Biet *et al*., 2014). Reducing the amount of specific amino acids in the diets extends lifespan in various species. The lifespan of *Drosophila* is extended by caloric restriction, but this effect is nullified when essential amino acids are provided (Grandison *et al*., 2009). In yeast cells, the amino acids threonine and valine activate Tor1 (the yeast orthologue of mTOR), promoting aging (Mirisola *et al*., 2014). Restriction of the essential amino acid methionine increases lifespan in rodents (Richie *et al*., 1994; López-Torres & Barja, 2008). Vascular aging is accelerated in rats fed a high methionine (2%) or high-protein (50%) diet (Fau *et al*., 1988). Alzheimer mice that undergo protein restriction cycles (a diet lacking essential amino acids is provided every other week), reduce IGF-1 levels, slow down the deposition of tau protein in the brain, and score higher on behavioral performance tests (Parrella *et al*., 2013).

How can we use these insights in the context of human diets? Meat is an important source of protein in the human diet in many cultures. Recent large studies show an association with meat intake and cardiovascular disease, type 2 diabetes, cancer, and all-cause mortality (Pan *et al*., 2012; Rohrmann *et al*., 2013; Ericson *et al*., 2013; Gonzalez & Riboli, 2010). However, the increased mortality due to meat intake cannot be solely attributed to the protein content of meat. Meat, and especially red processed meat, contains other ingredients that may have negative effects, such as specific fats, salt, or preservatives (Pan *et al*., 2012). Even so, a mounting body of research suggests a causal relationship between amino acid intake and a less optimal metabolism in humans. For example, a twofold rise in amino acid blood level leads to a 25% reduction in insulin sensitivity (Krebs *et al*., 2002) and directly infusing amino acids into the bloodstream increases insulin resistance in humans (Tremblay *et al*., 2007, 2005). Additionally, recent studies show an association between increased protein intake from meat and aging-related diseases in humans (Ericson *et al*., 2013; Levine *et al*., 2014). According to a study conducted in 6318 men and women from the NHANES III survey, people aged 50–65 years with a high protein intake (>20% of total calories) were four times as likely to die of cancer and had a 75% increase in mortality compared to people with a low protein intake (<10% of total calories). Participants with a low protein intake had also significantly lower IGF-1 levels. However, in this study, people over 65 may benefit from an increased protein intake (but only when their IGF-1 was also low). Yet, across all ages, there remained a fivefold increase in diabetes risk when comparing low with high protein intake. It is however important to point out that this study has its limitations: the sample size is rather modest, the food intake data are based on personal food questionnaires which are prone to underreporting, and the researchers do not substantially control for the overall balance of the diet, as people who eat much animal protein tend to consume less fruit or vegetables for example.

Interestingly, in this study, there was no association between *vegetable* protein intake and mortality (Levine *et al*., 2014). Other studies indeed corroborate that there is a difference between animal and vegetable protein in terms of health. Rats who are fed soy proteins instead of casein proteins have an increased lifespan (Iwasaki *et al*., 1988). In humans, proteins from vegetable sources induce IGF-1 less than proteins from animal sources (Allen *et al*., 2002). These physiological differences between animal and vegetable proteins can be explained by the fact that vegetable protein contains less methionine and sulfur-rich amino acids than animal protein. Substituting red meat with healthier (vegetable) proteins sources such as beans, lentils, tofu, and nuts could be recommended (Pan *et al*., 2012).

Some studies suggest that an increased protein intake can be protective in the elderly, reducing the risk of sarcopenia for example (Houston *et al*., 2008; Meng *et al*., 2009). While there is suggestive evidence that a higher protein intake can protect against sarcopenia in the elderly, there is no conclusive evidence that an increased protein intake reduces frailty or lowers overall mortality in older people (Pedersen & Cederholm, 2014). Interestingly, a low protein intake in the elderly leading to reduced muscle mass is accompanied by a reduction in IGF-1 (Castaneda *et al*., 2000). Animal and human studies show that reduced IGF-1 signaling leads to lower muscle mass, lower body weight, and a smaller phenotype but an increased lifespan, suggesting that decreased muscle mass is not always detrimental (Bartke *et al*., 2013; Holzenberger *et al*., 2003; Bonafè *et al*., 2003; Kojima *et al*., 2002; Pawlikowska *et al*., 2009; Suh *et al*., 2008; Guevara-Aguirre *et al*., 2011; Steuerman *et al*., 2011). Additionally, even if an increased protein intake should be beneficial in the elderly, recent studies suggest that a high protein intake in the nonelderly (<65 years old) can be detrimental, especially with regard to IGF-inducing animal proteins and especially in the long term (>15 years) (Levine *et al*., 2014). Many studies in humans that assess proteins intake and health are short- to medium-term studies.

While amino acids activate mTOR, there exists various substances that inhibit mTOR. Examples of such substances can be found in specific foods considered to confer health benefits, such as the ethylxanthine caffeine in coffee (Reinke *et al*., 2006; Wanke *et al*., 2008) and the polyphenol EGCG present in green tea (Van Aller *et al*., 2011), which reduce mTOR signaling. Quercetin, a substance found in fruits, vegetables, and spices, especially capers, lovage, and apples, is a mTOR inhibitor (Bruning, 2013). Resveratrol, a polyphenol found in the skin of red grapes also inhibits mTOR (Liu *et al*., 2010). It is important to note that many nutrients that confer potential health benefits do not solely affect mTOR, but often act on various aging-related pathways (see further on), including mTOR.

Other substances in food act downstream of the mTOR receptor. One of the consequences of too much mTOR activation is a reduction in autophagy, which leads to less degradation of protein aggregates in the cell. Protein aggregation is implicated in all kind of aging-related diseases, such as cardiomyopathy and Alzheimer's disease (Choi *et al*., 2013). Certain nutritional compounds, however, have the potential to attenuate protein aggregation, directly or indirectly. Curcumine, a substance found in the yellow spice turmeric, can directly inhibit the formation of amyloid-beta oligomers by interacting with the amyloidogenic regions of the amyloid-beta protein (Yang *et al*., 2005; Ono *et al*., 2004). Oleocanthal, a phenolic substance responsible for the bitter taste in extra-virgin olive oil, can reduce tau fibrillization (Monti *et al*., 2011) and enhance B-amyloid clearance *in vitro* and *in vivo*, potentially reducing the risk of Alzheimer's disease (Abuznait *et al*., 2013). Cinnamon extract can reduce B-amyloid formation and slow down cognitive deterioration in Alzheimer's disease model mice (Frydman-Marom *et al*., 2011).

## The IIS pathway, diets, and nutrition

Besides amino acids, nutrients like carbohydrates stimulate the IIS and mTOR pathway, especially via the insulin receptor, which activates both the mTOR and the IIS pathway (Um *et al*., 2006; Jewell & Guan, 2013). The glycemic index (GI) measures the area under the blood glucose curve after ingestion of a specific carbohydrate-containing food and indicates how much this food increases blood sugar and subsequently insulin production (Brouns *et al*., 2005). The glycemic load (GL) is a more precise measurement than the GI, because the GL takes into account both the GI and how much absorbable carbohydrates are contained in a specific food (Foster-Powell *et al*., 2002). Especially, bakery foods that contain an ample amount of carbohydrates combined with fats increase insulin production (Holt *et al*., 1997).

The IIS and mTOR pathway are two mechanisms through which high-glycemic index and glycemic load diets exert deleterious effects in the long term, because such diets deliver high amounts of nutrients in the form of easy-digestible carbohydrates, constituting a nutrient overload that activates aging-related nutrient-sensing pathways and leads to resistance of the insulin receptors (Um *et al*., 2006). Studies show that diets with a high-glycemic index and load increase the risk of various aging-related diseases such as type 2 diabetes, stroke, and heart disease (Beulens *et al*., 2007; Salmerón *et al*., 1997; Liu *et al*., 2000; Villegas *et al*., 2007; Sieri *et al*., 2013). On the other hand, some studies show no effect of glycemic index and load on type 2 diabetes (Mosdøl *et al*., 2007; Meyer *et al*., 2000).

Regarding weight loss, low-glycemic index diets are superior compared to low fat diets in terms of improving cardiovascular parameters and weight loss according to a Cochrane meta-analysis (Thomas *et al*., 2007). Adhering to a low-glycemic index diet improves metabolic parameters more than an isocaloric low-fat diet (Ebbeling *et al*., 2012). A strict hypocaloric low-glycemic index–load and mainly vegetable-based diet could even reverse diabetes in all subjects in 8 weeks time (Lim *et al*., 2011).

It should be noted that a large intake of fast-acting carbohydrates can have deleterious effects other than activation of the IIS pathway and mTOR pathway, for example via formation of advanced glycation end products (AGEs) (Singh *et al*., 2001). However, glycation and cross-linking are also well-known aging-mechanisms in the field of biogerontology (Vitek *et al*., 1994; Kilhovd *et al*., 1999).

Not only the macronutrient content (carbohydrates, proteins, or fats) of a diet, but also the micronutrient content can influence metabolism and more specifically insulin sensitivity. For example, blueberries contain anthocyanidins of which the intake is associated with improved insulin sensitivity and a decreased risk of type 2 diabetes (Muraki *et al*., 2013). Extracts from cinnamon can improve fasting blood glucose levels and lower glycated HbA1c concentrations in type 2 diabetes patients (Lu *et al*., 2012). Intake of cacao (the main ingredient of dark chocolate) is associated with increased insulin sensitivity and a reduced risk of cardiovascular disease (Buitrago-Lopez *et al*., 2011) and can slow down the progress of mild cognitive impairment in elderly people (Desideri *et al*., 2012).

## The role of fat and other nutrient-sensing pathways in aging

In this article, the role of protein and carbohydrates as macronutrients has been discussed. Fat constitutes a third important macronutrient. While amino acids and carbohydrates can directly activate aging-pathways such as mTOR and insulin/IGF-1, the role of specific proteins targeted by fatty acids and their potential role in aging is still unclear (Hou & Taubert, 2012; Hansen *et al*., 2013). However, mTOR and insulin/IGF-1 signaling play an important role in lipid metabolism during aging. Overstimulation of the mTOR and IIS pathway in preadipocytes by nutrients like carbohydrates and amino acids may contribute to their conversion into senescent-like preadipocytes which secrete pro-inflammatory compounds and induce insulin resistance in surrounding adipose cells. Additionally, senescent-like preadipocytes and insulin-resistant adipocytes are less efficient in handling and processing fatty acids, causing ectopic fatty acid deposition (in the liver, bone marrow, or blood vessels) contributing to heart disease, fatty liver disease, and stroke (Tchkonia *et al*., 2010; Stout *et al*., 2014).

As already stated in this paper, mTOR and insulin/IGF-1 signaling are prime examples of nutrient-sensing pathways involved in the aging process. Besides these canonical pathways, several other nutrient and energy sensing pathways are involved in the aging process. An example is the AMPK pathway, which is activated when the energy status in the cell is low (reduced ATP and increased AMP levels) (Houtkooper *et al*., 2010). Activated AMPK improves glucose uptake, mitochondrial biogenesis, and B-oxidation of fatty acids (Winder & Hardie, 1999). Other nutrient-sensitive modulators of the aging process are sirtuins (NAD-dependent histone deacetylases), which are activated by a low energy status of the cell (increased NAD+) (Houtkooper *et al*., 2012). Various nutrient-sensing pathways interact with each other. Mice overexpressing the sirtuin SIRT6 live longer and have reduced insulin and IGF-1 signaling (Kanfi *et al*., 2012), while AMPK signaling reduces mTOR (Alers *et al*., 2012) and works together with sirtuins to regulate energy metabolism (Price *et al*., 2012).

## Hormesis

Another interesting biogerontological insight is the concept of hormesis. Hormesis describes how small amounts of toxic substances can be beneficial because they induce cellular stress resistance mechanisms, such as upregulation of heat shock proteins, antioxidant enzymes, or phase II detoxification enzymes (Calabrese *et al*., 2007). For example, coffee contains slightly toxic compounds that activate Nrf-2, a transcription factor that upregulates cellular antioxidant proteins via the *antioxidant responsive element* (ARE) transcription factor (Boettler *et al*., 2011). Many vegetables and fruits contain hormetic substances that could have beneficial effects in the long term. Sulforaphane, a compound in cruciferous vegetables like broccoli, induces phase II detoxification enzymes (Hu *et al*., 2006), a process that can play a role in protecting brain tissue (Trinh *et al*., 2008) or reducing the risk of cancer (Hayes *et al*., 2008; Ambrosone *et al*., 2004). Hormesis can also explain why most antioxidants do not retard the aging process or increase lifespan (Bjelakovic *et al*., 2007). Large doses of extracorporeal antioxidants such as vitamin A, vitamin E, or beta-carotene downregulate cellular stress resistance proteins because high levels of antioxidants signal to the cell a reduced need for the production of antioxidant proteins and detoxification enzymes, which are in fact much more effective than extracorporeal antioxidants to provide resistance against cellular stress.

## Caloric restriction and fasting

Besides specific macronutrient ratios and micronutrient intake, *less* nutrients could also reduce the risk of aging-related diseases. Caloric restriction and fasting drastically reduce IIS and mTOR signaling and increase lifespan in many species, ranging from worms to rhesus monkeys (Fontana *et al*., 2010; Colman *et al*., 2009). A long-term study (>20 years) at the Wisconsin National Primate Research Center showed that age-related mortality was threefold lower in the caloric-restricted rhesus monkeys (Colman *et al*., 2009). However, another long-term study conducted by the National Institute of Aging (NIA) in caloric-restricted rhesus monkeys did not show an increase in lifespan (Mattison *et al*., 2012). This different outcome could be because in the NIA study, the control group food rations had a different dietary composition or because the control group was not fed *ad libitum* and as a consequence was also subjected to a minor form of caloric restriction (Partridge, 2012). This latter explanation is supported by the fact that the control monkeys in the NIA study weighted substantially less than the national average for body weight in the same age groups (Colman *et al*., 2014). However, the question remains if *ad libitum* feeding is more an obesity model than a normal feeding state in primates, implying that caloric restriction would ‘normalize’ extra food intake in primates rather than further improving health and lifespan in primates with a normal food intake. Additionally, other factors besides diet such as the genetic background of the test subjects or the experimental design may also account for these discrepancies.

In humans, long-term caloric restriction is associated with improved cardiometabolic parameters and a reduction of atherosclerosis and diastolic dysfunction (Fontana *et al*., 2004; Meyer *et al*., 2006; Heilbronn *et al*., 2006). Intermittent fasting (also referred to as ‘low level caloric restriction’) could be a more achievable alternative to caloric restriction. Fasting every other day or on several days per week (during which one consumes up to 75% less calories) leads to a reduced total caloric intake in the long term. Preliminary studies show improvement of various cardiovascular markers such as LDL cholesterol and insulin sensitivity (Varady *et al*., 2009; Halberg *et al*., 2005). Fasting could be especially beneficial in the treatment of cancer, as fasting can make tumor cells more vulnerable to chemotherapy and improve regeneration of the immune system after chemotherapy (Raffaghello *et al*., 2008; Lee *et al*., 2012; Cheng *et al*., 2014). Further research is needed to investigate the long-term effects of fasting and caloric restriction on aging-related diseases in humans.

## Discussion

Insights from biogerontology can be very useful to assess the efficacy of diets that aim to reduce the risk of aging-related diseases, or mitigate them. Without biogerontology as a benchmark or touchstone, one has to rely mainly on clinical trials which focus on short- or medium-term primary outcomes, like reducing LDL cholesterol or triglycerides, but do not take into account long-term outcomes, like an increased (healthy) lifespan. A thorough understanding of the aging process can help to better predict the efficacy of diets and specific nutritional interventions. For example, biogerontological knowledge predicts that diets being high in animal protein (like the Atkins diet or some interpretations of the paleo diet) are probably not healthy in the long term and that taking antioxidant dietary supplements does not increase lifespan. On the other hand, specific diets that have been shown to reduce the risk of various aging-related diseases seem to act via important biogerontological pathways and mechanisms.

For example, a Mediterranean diet can substantially reduce the risk of cardiovascular disease, and this to a greater extent than a standard low-fat diet (De Lorgeril *et al*., 1994). The Mediterranean diet is a low-glycemic index diet that contains many mithormetic, anti-amyloidogenic, and anti-inflammatory compounds found in vegetables, fruits, red wine, olive oil, or nuts. In Okinawa, where substantially more centenarians live with often good health, there exists a tradition of adhering to a vegetable-rich low-glycemic index diet and practicing a form of minor caloric restriction (Okinawans tend to consume small portions) (Willcox *et al*., 2006, 2007). Vegetarian diets are associated with a reduced all-cause mortality (Orlich *et al*., 2013). Compared to meat-containing diets, a vegetarian diet provides more plant-based proteins and a lower methionine content, which could contribute to the increase in lifespan and health (McCarty *et al*., 2009; Cavuoto & Fenech, 2012).

In the future, even healthier diets could be devised using biogerontological insights. These diets would not overstimulate mTOR, insulin, and IGF-1 receptors, would deliver mithormetic substances that stimulate cellular stress resistance pathways and would provide a copious supply of substances that beneficially influence myriad mechanisms involved in the aging process such as protein/amyloid aggregation, autophagy, lipid peroxidation, and mitochondrial dysfunction. A synthesis of biogerontological, nutritional, and medical knowledge can be framed in a new field called ‘nutrigerontology’. Nutrigerontology can be defined as the scientific discipline that studies the impact of nutrients, foods, macronutrient ratios, and diets on lifespan, the aging process, and aging-related diseases.

The goal of nutrigerontology is to research and use compounds, foods, and diets that can reduce the risk of aging-related diseases and increase the (healthy) lifespan. Some interventions can slow down the progress of these aging-related diseases or even reverse them, like type 2 diabetes or cardiovascular disease (Lim *et al*., 2011; De Lorgeril *et al*., 1994). While biogerontology mainly focuses on unraveling the mechanisms of aging and gerontology also focuses on the social and psychological aspects of aging, nutrigerontology signifies a practical approach. Nutrigerontology will be mainly practiced by physicians (cardiologists, endocrinologists, oncologists, neurologists, family physicians) and dieticians. Nutrigerontology can be applied in many different medical fields where aging-related diseases are common, such as cardiology (atherosclerosis, diastolic dysfunction, ventricular hypertrophy, high blood pressure), endocrinology (type 2 diabetes), neurology (Alzheimer's disease, stroke, Parkinson's disease), ophthalmology (cataract, aging-related macular degeneration), oncology (cancer), or rheumatology (osteoporosis, sarcopenia, frailty). While geriatrics is the medical discipline that treats the elderly, nutrigerontology is a medical and scientific discipline that wants to act as much as possible on the period before people are old, frail, and afflicted by a myriad of aging-related diseases.

Medical students should be taught about nutrigerontology and learn how various diseases that afflict our society are in fact (accelerated) aging-related diseases. They will learn how evidence-based long-term dietary interventions can reduce the risk of these diseases and mitigate them, slow down their progress or revert them. In a nutrigerontology course, students will be instructed in the aging process and how mechanisms of aging cause many different aging-related diseases. This is important, as addressing and eliminating only one disease of aging, like cardiovascular disease for example, would only add 2,87 years to average lifespan (Barzilai & Rennert, 2012), because patients would still be afflicted by other diseases of aging, like Alzheimer's disease, diabetes, or cancer. Therefore, it would be paramount to address aging itself, and not only specific aging-related diseases without emphasis on aging (biogerontology) and relevant interventions (nutrigerontology).

However, extrapolating knowledge from the biogerontological field to nutritional science and medicine has to be performed carefully, as many model organisms used in aging research (yeast, fruit flies, or mice) are much shorter lived than humans and differ from humans genetically and physiologically and may have been exposed to different mechanisms of natural selection (more selection for fast reproduction in mice for example). On the other hand, the conservation of many aging-related pathways such as mTOR and insulin/IGF-1 in evolutionary very different organisms over millions of years strongly suggests that such pathways are also relevant in humans. Interpretation of study results from model organisms is sometimes challenging, because many confounding factors can influence lifespan in laboratory animals, such as genetic differences between wild-type or laboratory strains, laboratory environment (food supplies, husbandry, bedding, litter mates), and accidental or premature death (Partridge & Gems, 2007).

There is an urgent need of integrating knowledge from the biogerontological field with nutritional science and medicine in view of the increasing epidemic of aging-related and metabolic diseases, driven by a rapidly aging population and an unhealthy lifestyle (Anon, 2011). In 2010, an estimated 524 million people were 65 years or older (8% of the world population). By 2050, this number will increase to about 1.5 billion (16% of the world population). In 2008, noncommunicable aging-related diseases such as heart disease, Alzheimer's disease, and cancer constituted 86% of the burden of disease in high-income countries. Dementia alone accounted for 600 billion dollar in healthcare costs worldwide. From the age of 65, the risk of dementia doubles every five years resulting in 30% of people between 85–89 years being afflicted with dementia (Anon, 2011).

A better understanding of the aging process and ensuing practical, clinical, and nutritional interventions could not only improve the quality of life and health of many people, but would also reduce aging-related healthcare costs and long-term care expenditures, increase potential working span and productivity, and strengthen economical competiveness.

Both animal and human research into aging and nutrition should be promoted, so that various ways of how nutrients, foods and diets interfere with the aging process can be researched. Examples of such studies are protein restriction cycles that reduce the progress of Alzheimer's disease in mice or dietary patterns that occur more in centenarian populations (De Lorgeril *et al*., 1994; Willcox *et al*., 2007). In light of our aging population and the concomitant rise of aging-related diseases, a new interdisciplinary and practically orientated field like nutrigerontology is urgently needed.

## Funding

No funding information provided.

## Conflict of interest

The author has nothing to disclose.
